# Cardiac Surgery and Transcatheter Intervention for Valvular Heart Disease in Carcinoid Syndrome: Risk Factors, Outcomes, and Evolving Therapeutic Strategies

**DOI:** 10.3390/jcdd11110359

**Published:** 2024-11-07

**Authors:** Mariagrazia Piscione, Valeria Cammalleri, Giorgio Antonelli, Valeria Maria De Luca, Myriam Carpenito, Dario Gaudio, Nino Cocco, Antonio Nenna, Carmelo Dominici, Antonio Bianchi, Francesco Grigioni, Gian Paolo Ussia

**Affiliations:** 1 Department of Medicine and Surgery, Università Campus Bio-Medico di Roma, Via Alvaro del Portillo, 21, 00128 Rome, Italy; mariagrazia.piscione@unicampus.it (M.P.); giorgio.antonelli@unicampus.it (G.A.); valeriamaria.deluca@unicampus.it (V.M.D.L.); dario.gaudio@unicampus.it (D.G.); f.grigioni@policlinicocampus.it (F.G.); gpussia@hotmail.com (G.P.U.); 2 Unit of Cardiovascular Science, Fondazione Policlinico Universitario Campus Bio-Medico, Via Alvaro del Portillo, 200, 00128 Rome, Italy; m.carpenito@policlinicocampus.it (M.C.); n.cocco@policlinicocampus.it (N.C.); 3 Department of Cardiovascular Surgery, Università Campus Bio-Medico di Roma, Via Alvaro del Portillo, 21, 00128 Rome, Italy; a.nenna@policlinicocampus.it (A.N.); c.dominici@policlinicocampus.it (C.D.); 4 Internal Medicine, Endocrinology and Diabetes Unit, Fondazione Policlinico Universitario Agostino Gemelli IRCCS, Department of Medical and Surgical Sciences, Catholic University of the Sacred Heart, 00168 Rome, Italy; antonio.bianchi@policlinicogemelli.it

**Keywords:** carcinoid heart disease, surgical valve replacement, transcatheter edge-to-edge repair

## Abstract

Carcinoid heart disease (CHD) affects right-sided valves and causes significant mortality and morbidity. Even though the pathophysiology of the disease is not entirely understood, it is known that chronic exposure to high levels of circulating serotonin is the main factor responsible for developing valvular heart disease. Cardiac imaging plays a critical role in the management of CHD, so the final diagnosis can be performed through multimodal imaging techniques and the measurement of biomarkers. Moreover, in observational studies, surgical treatment of carcinoid-induced valve disease has been found to improve outcomes. Despite advancements in pre-operative preparation in recent years, mortality rates remain high in elderly patients and those with multiple comorbidities due to the risk of intra-operative carcinoid crisis and high post-operative bleeding. In this comprehensive review, we will analyze the causes of carcinoid syndrome and how it can result in severe right heart failure. The role of different imaging modalities in detecting heart valve disease will be discussed together with the therapeutic options at our disposal, such as medical treatment, surgery, and the novel role of transcatheter intervention.

## 1. Introduction

Neuroendocrine tumors (NETs) are rare types of neoplasms [1]. Carcinoid tumors, a subset of NETs that most commonly arise from the distal small intestine and proximal colon, affect approximately 1 in 75,000 people. Notably, only about 50% of patients with carcinoid tumors go on to develop carcinoid syndrome (CS). The heart is involved in 50% of cases, resulting in commonly represented and diagnosed carcinoid heart disease (CHD) [2]. CS is a condition that predominantly manifests in patients with liver metastases, as the hepatic vein transports vasoactive substances generated by the primary tumor or metastases and releases them into the systemic circulation without pulmonary inactivation [3,4]. CS is characterized by episodes of vasomotor changes such as flushing and hypotension, diarrhea, and bronchospasm [5]. CHD usually involves the right-sided heart valves and leads to right heart failure (RHF). A 60% male preponderance has been documented, with a mean age range of 56–63 years at the time of diagnosis [5]. The early diagnosis and the correct choice of the most suitable option for valve treatment are the main issues in CHD. Indications for valve surgery in CHD patients are explained in a consensus statement, including progressive RHF with echocardiographic findings of moderate to severe insufficiency of the right-sided valves [1]. Patients with CHD are generally older and frailer, so therapeutic options have to be patient-tailored and carefully chosen. This review provides an analysis of CHD, focusing on its impact on heart valves and progression to RHF. We discuss the diagnostic role of cardiac imaging and biomarkers, as well as current therapeutic strategies, including medical management, surgical valve replacement, and transcatheter interventions. By offering insights into optimal treatment approaches and early intervention, this review aims to guide clinicians in managing CHD and contributes to advancing knowledge in the field.

## 2. Pathophysiology of Carcinoid Heart Disease

The pathogenesis of CHD is not completely understood, as the role of numerous vasoactive substances secreted by the tumor in causing tissue damage is still unclear. NETs release serotonin and other peptides as growth factors. They do not act independently, and crosstalk between signaling pathways likely plays a crucial role in the pathophysiology of the fibrotic process [6]. It is hypothesized that NETs produce prostaglandin, histamine, bradykinin, serotonin or 5-hydroxytryptamine (5-HT), tachykinins (substance P, neurokinin A, neuropeptide K), and transforming growth factor-β. These, in turn, stimulate fibroblast growth and fibrogenesis and enhance oxidative stress [7].

It has been widely demonstrated that one of these substances, 5-HT, has a pivotal function in the pathogenesis of CHD [7,8]. There is evidence that the 5-HT2B receptor subtype, expressed on the surface layer of cardiac valves, has a leading role in the progression of the disease since, in 90% of cases, right heart valves are involved [8].

The consequence of releasing these mediators and pro-inflammatory cytokines is the accumulation of plaque-like material, constituted of myofibroblasts, smooth muscle cells, and extracellular components such as collagen, myxoid matrix, and elastin [9]. These are firstly stored within the intima of the pulmonary arteries, the aorta, and the venae cavae and then within the endocardial surfaces of valve leaflets, the subvalvular apparatus constituted of chordae tendineae, papillary muscles, and the cardiac chambers. Moreover, fibrotic depots can be found on the pulmonary arterial side of the pulmonary valve (PV) and ventricular leaflets of the tricuspid and PVs, respectively [10]. Additionally, coronary artery vasospasm and ventricular tachycardia have been reported as being among the cardiac effects of 5-HT [11,12,13,14].

## 3. Clinical Outcomes of Carcinoid Heart Disease

CHD is the major source of morbidity and mortality for patients with CS, due to progressive dysfunction of the valves involved (mostly tricuspid and PV) and decompensated heart failure in the advanced stage of the disease [1]. The 3-year survival rate of patients with CS and CHD is poor, at 31%, compared to 68% for those without heart involvement [15]. Nevertheless, patient outcomes have significantly improved in recent years due to the introduction of new antitumor medical therapies, new cardiac imaging technologies, perioperative management, and advancements in surgical and transcatheter interventions for valve defects [16]. However, due to the nonspecific or even absent cardiac symptoms in the early stages, diagnosing CHD may be delayed without routine echocardiographic screening. Most patients with significant valve involvement, particularly affecting the tricuspid and pulmonary valves, initially exhibit only mild symptoms, such as shortness of breath during moderate exertion [17]. Pulsatile hepatomegaly, peripheral edema, ascites, and elevated jugular venous pressure are manifestations of the advanced phase of CHD [17]. If left untreated, these conditions can progressively worsen and eventually lead to advanced RHF due to the maladaptation of the right ventricle (RV) to pressure or volume overload (secondary to pulmonary stenosis or tricuspid regurgitation, respectively) [18]. Coronary artery vasospasm can occur in CHD people with non-obstructive coronary artery disease since vasospasm is linked to serotonin-induced vasoconstriction in the setting of endothelium dysfunction [13,14]. Furthermore, serotonin may induce paroxysmal ventricular tachycardias and atrial arrhythmias [14]. While the development of coronary vasospasm or ventricular tachycardia is unpredictable, valve disease can be clinically suspected, correctly diagnosed, and promptly treated [19,20]. The heart involvement can progress rapidly, potentially leading to decompensated heart failure if not managed in a timely manner.

## 4. Biomarkers for Screening and Diagnosis

Early diagnosis of CHD is crucial, and the development of a biochemical marker that is both sensitive and specific in predicting the presence and severity of CHD could allow for a more targeted use of echocardiography [1]. Elevated serum chromogranin A (CgA) levels proved to be a useful diagnostic marker for NETs, although the accuracy of serum CgA varied depending on the specific type of NET [21]. Furthermore, CgA seems to not be a reliable indicator of recurrences, and a false increase in this biomarker can be common in the context of atrophic gastritis or after proton pump inhibitor treatment and renal and liver diseases [22,23]. 5-Hydroxyindoleacetic acid (5-HIAA) is a byproduct of serotonin breakdown that can be detected in serum or urine samples. 5-HIAA has demonstrated excellent sensitivity for CHD, but it lacks specificity [24]. Similarly, Activin A levels ≥0.34 ng/mL were found to have a high sensitivity but a rather low specificity for detecting CHD [1]. Wedin et al. showed that liver metastases from CHD and liver tumor burden were significantly correlated with serum 5HIAA, indicating that the biomarker turns positive in the advanced stages of the disease, even if there was no clear correlation with changes in the disease status [25]. N-terminal pro-B-type natriuretic peptide (NT-proBNP) is the most valuable and commonly used biomarker demonstrating a prognostic and diagnostic value in relation to cardiac involvement [21,22,23]. Levy et al. produced the largest validation study for biomarkers in CHD, suggesting that NT-proBNP levels higher than 6.5 pmol/L should still be considered for CHD screening when there is clinical suspicion. Patients whose NT-proBNP is higher than 27 pmol/L ought to have even closer monitoring for a high risk of CHD development [26]. Currently, other biomarkers are under investigation for CHD screening and prognosis in patients suffering from NETs [27]. Recently elevated soluble suppression of tumorigenesis-2 (sST2) levels were correlated with more severe heart valve dysfunction and signs of cardiac fibrosis. Plasma levels higher than 35 ng/mL correlated with worse outcomes [27].

## 5. Cardiac Imaging in Carcinoid Heart Disease

**Figure 1:**
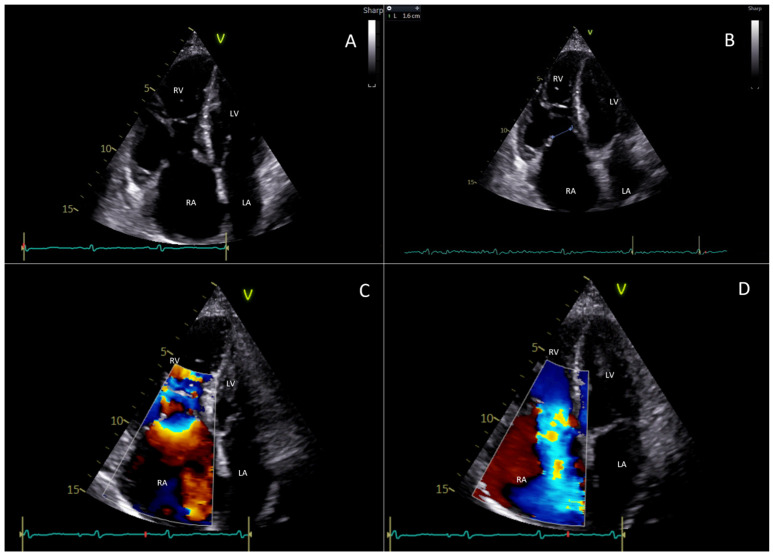
This figure represents a case evaluated at Fondazione Policlinico Universitario Campus Bio-Medico, Rome. It is about a 77-year-old woman who suffers from carcinoid heart disease with involvement of the right heart chambers and tricuspid valve. (**A**) The transthoracic echocardiogram (4-chamber view) shows a significant dilatation of right heart chambers with “bulging” of the interventricular septum; the right papillary muscles, chordae tendineae, and tricuspid valve leaflets appear thickened; a diastolic restricted opening of the tricuspid valve is also appreciable. (**B**) The transthoracic echocardiogram (4-chamber view) in systole shows severe malcoaptation between the tricuspid septal and anterior leaflets creating a gap of 1.6 cm. (**C**) The color Doppler flow detects a stenotic filling in diastole. (**D**) The color Doppler in systole shows torrential tricuspid regurgitation. LA: left atrium; LV: left ventricle; RA: right atrium; RV: right ventricle.

CHD assessment and disease severity evaluation is primarily performed with transthoracic echocardiography (TTE) [16]. A wide variety of echocardiographic findings characterize heart involvement differently in early and advanced disease [16]. The severity of tricuspid valve (TV) dysfunction can range from mild, isolated thickening of a single valve leaflet, without a significant reduction in leaflet mobility, to advanced retraction and immobility of multiple leaflets. The onset of valvular regurgitation and stenosis represents the initial phase of the disease since leaflets are less mobile and do not close in systole, resulting in a frequent central gap, and partially open during diastole, resulting in restricted mobility (Type IIIa in the Carpentier Classification) [16,28,29]. Moreover, their typical concave curvature is reduced, the leaflet dynamic motion is modified during the diastole, and they exhibit a rigid, board-like motion. As the disease progresses, the valve leaflets gradually thicken, with the chordae and papillary muscles also being affected. When conditions become extremely severe, leaflets become rigid, retract, and cease to coapt so that functionally, massive/torrential tricuspid regurgitation unifies the RV and atrium into a single chamber. All these anatomical findings can be easily studied by a comprehensive TTE using parasternal, apical, and subcostal standard views [30]. Severe tricuspid regurgitation is also indicated by a Doppler profile consisting of a triangular shape (sometimes called “dagger-shaped”), due to the rapid decrease in the driving pressure during systole caused by an increase in right atrial pressure [28]. Figure 1 illustrates the typical echocardiographic findings of TV involvement in the setting of CHD.

**Figure 2:**
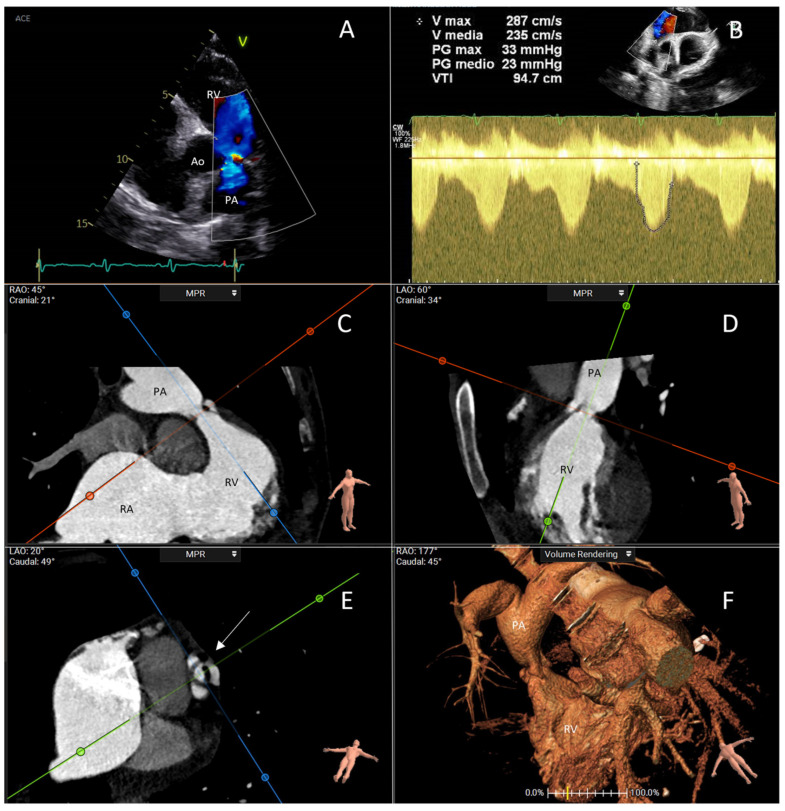
This figure represents a case evaluated at Fondazione Policlinico Universitario Campus Bio-Medico, Rome. It is about a 51-year-old woman who had severe pulmonary valve stenosis and torrential tricuspid regurgitation in the setting of carcinoid heart disease. (**A**) The transthoracic echocardiogram with color Doppler shows turbulence in blood flow across the stenotic pulmonary valve. (**B**) Continuous-wave Doppler examination shows a typical curve of severe pulmonary stenosis with increased gradient across the valve; this was a case of low-flow–low-gradient pulmonary stenosis because of the coexistence of torrential tricuspid regurgitation. (**C**–**E**) CT scan examination with multiplanar reconstruction in systole reveals the morphology of the pulmonary valve (arrow), showing thickened and hypomobile cusps. (**F**) Three-dimensional volume rendering showing the narrowed pulmonary valve and the post-stenotic dilation of the pulmonary artery. Ao: aorta; PA: pulmonary artery; RA: right atrium; RV: right ventricle.

The PV is less affected compared to the TV, with only 69% of patients exhibiting PV steno-regurgitation [16]. In CHD, changes in PV morphology and mobility are similar to those seen in the TV, with the valve annulus thickened and the pulmonary cusps failing to coapt. However, the presence of PV involvement may go unnoticed, since this valve is challenging to visualize in parasternal and subcostal RV outflow views [30,31]. Moreover, the stenotic gradients and the regurgitant volume of the PV may be underestimated by concomitant torrential tricuspid regurgitation since the stroke volume reaching the PV is reduced [32]. Furthermore, the cut-off values for vena contracta in detecting pulmonary regurgitation have not been validated in adults with CHD and reduced RV stroke volume, potentially leading to underestimation of regurgitation severity when using standardized indices [32,33]. Figure 2 illustrates the typical echocardiographic and computed tomography (CT) scan findings of PV in CHD.

Since not all the echocardiographic findings may be clearly visible on 2D TTE, advanced echocardiographic techniques such as 3D TTE may be useful in identifying and assessing valve pathology. Due to the anterior position of the right heart, 3D TTE is able to provide images of equal or even better quality in comparison to 2D transesophageal echocardiogram (TEE), making it a valuable alternative for detailed right heart and valve assessment [30]. In these cases, 3D imaging enhances visualization of leaflet involvement and the subvalvular apparatus, improving diagnostic accuracy and aiding in treatment planning [30]. Additionally, a 3D approach can provide valuable assistance in quantifying both tricuspid and pulmonary regurgitation by measuring the 3D vena contracta area, as well as in assessing tricuspid and pulmonary stenosis through 3D planimetry of the valve opening orifice area [30]. The TEE may provide additional high-resolution images that allow for detailed assessment of valve leaflets and the subvalvular apparatus, which are commonly affected by carcinoid-related plaque deposits [30]. TEE examination of the TV should include imaging from several depths, multiplane angles, and 3D acquisitions [30].

The echocardiographic study in CHD patients must always include the analysis of morphology and function of the RV [33,34]. In addition to the standard parameters for assessing the dimensions and function of the RV (such as RV basal, midventricular diameter, length, TAPSE, FAC, and TDI S’), advanced echocardiographic assessment (including RV free-wall longitudinal strain and 3D volumes) should be always performed [33,34]. Interestingly, RV strain is slightly decreased even in patients with elevated 5-HIAA levels, despite the absence of clear involvement of the right heart chambers and valvular heart disease [33]. In this setting, cardiac magnetic resonance (cMR) may be helpful in the quantification of valvular vices, detection of myocardial fibrosis, and extension of myocardial metastases into extracardiac structures [32,35,36,37]. Calcifications of affected valves are rare and may be considered a notable negative echocardiographic feature of CHD, which may be further evaluated through cardiac CT [36]. Moreover, CT is highly accurate in measuring the regurgitant orifice area and the planimetric area of the TVs and PVs, particularly in cases of multi-valvular disease when there is a risk of underestimation and in the case of transcatheter management [36].

## 6. Carcinoid Disease of the Left Sections of the Heart

The preferential right heart involvement is most likely related to the inactivation of the vasoactive substances by the lungs [1]. In 5–10% of patients with left-sided valvular pathology, extensive liver metastases, a bronchial carcinoid, or a patent foramen ovale (PFO) should be suspected [1]. In the presence of a bronchial carcinoid, PFO with right-to-left shunt, and high circulating levels of vasoactive substances that surpass the hepatic and pulmonary degradative capacity, left-sided disease may manifest in as many as one-third of cases [37]. In the study of Bhattacharyya et al., 30% of patients suffered from CHD with left heart involvement [16]. While in 87% of the cases it was associated with a PFO, the remaining patients had multiple bronchial metastases [16]. According to the high prevalence of PFO in the CHD population, it is hypothesized that PFO may open when right atrium pressures increase [38]. Before surgical treatment of right heart valves, it is critical to perform an evaluation with a TTE. Douglas et al., in 2023, reported a high incidence of PFO in CHD patients who were referred to their center for surgery of right-sided disease. Furthermore, they demonstrated how the risk of hemodynamic instability may be reduced by using an early percutaneous PFO closure before the surgical procedure instead of during the open valve surgery [38].

## 7. Physiopathology of the Development of Right Heart Failure in Carcinoid Heart Disease

The mechanism of the development of RHF in CHD is not entirely understood. The major causes of RHF can be summarized as reduced myocardial compliance, excessive preload, and increased afterload [39]. In CHD, the plaque-like fibrotic lesions resulting from the proliferation of myofibroblasts and deposition of extracellular matrix may cause adverse RV remodeling and subsequent increased intraventricular pressure [40]. The involvement of the PV with stenosis leads to RV pressure overload, prompting an initial “adaptive” remodeling response in the RV, characterized by relatively preserved volumes and function, along with compensatory “concentric” hypertrophy [18]. Over time, if adrenergic tone and neurohormonal activation occur, RV contractility decreases, and afterload continues to rise, causing the RV to dilate further in an effort to maintain stroke volume (heterometric adaptation), leading to the onset of signs and symptoms of severe RHF [18]. The involvement of the tricuspid apparatus causing severe tricuspid regurgitation is also common in CHD. Volume overload is in general more tolerated than pressure overload by the RV even though it causes a septal shift leftward altering the interventricular dependence and both RV and LV contractility [18,41]. In conclusion, the concomitant presence of pressure and volume overload together with the elevated intraventricular pressures determine abnormalities of excitation–contraction coupling and the severe deterioration of the RV function, which also may suffer from concomitant pericardial constriction [42].

## 8. Treatment

Patients who receive the diagnosis of CHD and have metastatic NET should be managed by a multidisciplinary team consisting of endocrinologists, oncologists, cardiologists, pathologists, and cardio-surgeons who have experience in managing this condition so that they operate in specialized centers. In the absence of early intervention, NET patients with CHD will inevitably suffer from a substantial reduction in life expectancy (of 30% according to Møller et al. [15]) and progressive RHF [43].

### 8.1. Treatment of Right Heart Failure

Symptomatic CHD associated with RHF primarily develops from compromised TV and PV function. The medical management to alleviate symptoms consists of preload optimization, RV afterload reduction, and RV contractility enhancement [44]. Patients with chronic RHF generally benefit from volume removal and decongestion [44]. Loop and thiazide diuretic agents are suggested in addition to aldosterone antagonist therapy [44]. Reducing the preload can lead to a decrease in TV annular dilatation and regurgitation, as well as RV wall stress and septal deformation. However, it has to be stated that an excessive depletion of intravascular volume may result in a further decline in cardiac output, thereby inducing fatigue and dyspnea [45]. A reduction in RV afterload is most effectively reached by optimizing medical therapy and restoring left-heart filling pressures to normal levels. Both an increase in pulmonary vascular compliance and a decrease in pulmonary vascular resistance may result from a decrease in left atrial pressure [46]. If inotropic support is needed to maintain cardiac output, dobutamine and milrinone may be used carefully to avoid vasodilatation and hypotension [47].

### 8.2. Treatment of Carcinoid Syndrome Symptoms

Somatostatin analogs are used for symptom management in patients diagnosed with CS [48]. Their antiproliferative potential has led to their growing utilization in asymptomatic patients because of their impact on progression-free survival and on the progression of the disease [48]. It has been demonstrated that treatment with somatostatin analogs, such as ocreotide and lanreotide, which decrease circulating tumor metabolites (5-HT), can induce a biochemical and symptomatic response in 0% to 70% of patients [48,49]. Another drug that may be used to treat NETs associated with CS in Europe is Interferon-alpha. In patients with negative somatostatin receptor status, it stimulates T lymphocytes and reduces tumor size with evidence of survival benefit [50]. The estimated subjective response rate is around 60%, while biochemical responses are detected in 40–50% of the patients [50]. When treating metastatic NETs, peptide receptor radionuclide treatment (PRRT) is a viable therapeutic option [51]. The idea is to administer tumoricidal radiation to the tumoral site with a similar targeting molecule labeled with a diagnostic radionuclide. Actually, Zandee et al. have demonstrated that PRRT with [Lutetium-177-DOTA0-Tyr3] octreotate (177Lu-DOTATATE) results in increased quality of life in patients with NETs [52]. Moreover, Telotristat ethyl (inhibitor of an enzyme involved in serotonin synthesis) has recently been approved to treat diarrhea in CHD patients [53]. Generally, carcinoids are not responsive to chemotherapy, so in patients with liver metastases, transcatheter arterial embolization may be considered an effective treatment option for diminishing NET burden and, consequently, hormone levels [54], but detrimental effects such as bleeding or liver failure may occur [55].

### 8.3. Surgical Management of Valve Disease

In the setting of surgical management of the TV regurgitation, valve repair over the valve replacement is generally suggested, except for Ebstein’s anomaly and CHD [56]. During the same procedure, PV can also be substituted. Somatostatin analog pre-treatment to manage carcinoid hormonal activity and pre-operative optimization of nutritional status are crucial for determining the appropriate timing of surgery. Research suggests that outcomes depend on earlier intervention and the degree of RV dysfunction [56], Møller et al. have demonstrated the prognosis for patients with CHD has improved throughout the previous 20 years, relating to a prompter surgical replacement of valves so that RHF does not develop [16]. Additionally, it is imperative to safeguard the RV function during the procedure, as the RV is often already dilated in most surgical candidates and vulnerable to dysfunction [57]. Recently, after valvular surgery, symptoms significantly improved in 75% of patients with symptomatic CHD, according to a study conducted by Connolly et al. [58].

TV regurgitation is the predominant lesion in cases of CHD, leading to higher mortality rates than carcinomatosis [59]. Therefore, unless surgery is contraindicated, TV replacement is recommended in patients with symptoms of RHF, progressive right heart dilation or enlargement, and before hepatic surgery. [20,60]. The literature reports several cases of TV, isolated or not, operated on in patients with CHD. Specifically, the Mayo Clinic’s 30-year experience is the most valuable one, and Nguyen et al. demonstrated that early mortality decreased from 29% between 1985 and 1994 to 7% between 1995 and 2004 and was currently at 5% [60,61]. Moreover, in patients with CHD and hepatic metastases who are at elevated risk of hemorrhage during surgery due to impaired liver function, venous stasis, and oncological status, it is crucial to prioritize hemorrhage control, utilize autologous blood recovery systems, and optimize post-operative coagulation through point-of-care techniques. [61,62]. So, since the modification of perioperative somatostatin analog protocols and pre-operative care has reduced in-hospital mortality, CHD surgery remains the best choice for younger patients in relatively better health. Actually, PV replacement with RV outflow tract patch enlargement is performed to manage PV steno-regurgitation and alleviate RV outflow tract obstruction [62]. In cases where the surgical risk is too high, patients with stenotic TVs or PVs have experienced symptomatic improvement after balloon valvuloplasty; however, recurrence of symptoms has been documented [1]. The selection of valve prostheses remains a matter of debate. The prosthesis should be tailored for each patient, considering factors such as future therapeutic interventions, life expectancy associated with the malignancy, and the risk of bleeding [1]. The implantation of a mechanical prosthesis requires anticoagulation therapy, which raises the bleeding risk in patients with liver metastases. Additionally, mechanical prostheses placed in the tricuspid position increase the risk of thrombosis [1]. For these reasons (the need for anticoagulation and the risk of thrombosis), bioprosthetic valves are generally preferred since the valves of a more recent generation are the most resilient [1]. Furthermore, optimizing CS treatment following cardiac surgery may support bioprosthetic valves instead of mechanical prostheses to counter the detrimental consequences of vasoactive substances [20]. Even if periprocedural deaths have decreased in the last years, surgical valve therapy may cause serious complications such as vasoplegia, RHF, and perioperative coagulopathy [63,64]. Consequently, survival rates after valve surgery remain low, particularly in frail patients [64,65]. Despite that, currently, there are no satisfactory risk stratification models for predicting the outcomes of surgery. However, older patients with advanced neoplasms and RHF may theoretically benefit the most from transcatheter-based procedures.

### 8.4. The Role of Transcatheter-Based Procedures

The high mortality and morbidity associated with cardiac surgery in patients with CHD have led to the development of transcatheter techniques. Percutaneous PV implantations have been performed on patients diagnosed with NETs who also present with dysfunctional bioprosthetic PVs and TVs [66]. Worldwide, five PVs have been implanted de novo with a catheter-based technique [67,68,69,70,71,72]. Our center’s experience has demonstrated that PV transcatheter implantation is a reduced-risk procedure able to improve the patient’s quality of life in a short follow-up [72]. Similarly, percutaneous catheter-based interventions such as edge-to-edge repair, direct annuloplasty, or valve replacement are innovative and minimally invasive alternatives to manage severe tricuspid regurgitation in high-risk patients who are unable to undergo open surgery due to their performance status and comorbidities [66]. When considering transcatheter procedures, proper planning of valve treatment priorities is essential, and the anatomy and mechanisms of the valvular defects must be thoroughly investigated.

In addition to the aforementioned echocardiographic assessment (both TTE and TEE), cardiac CT plays a crucial role in the selection of patients for TTVI by providing detailed anatomical information on the TV and surrounding structures, ensuring proper device sizing and optimal procedural planning [73,74]. In particular, the edge-to-edge repair technique, which aims to enhance the coaptation of leaflets in the regurgitant TV, required a detailed echocardiographic screening and not a CT scan evaluation [75]. The unfavorable anatomical conditions for tricuspid edge-to-edge repair procedure in CHD are the stenosis of the native valve and the reduced leaflet lengths, which cause an elevated coaptation gap. Leaflet thickening or shortening, a typical characteristic of TV affected by CHD, results in a relative pathoanatomic contraindication [75]. The tricuspid annuloplasty decreases the annular diameter in TV regurgitation by transfemoral rings or sutures. A detailed echocardiographic and CT study is required [75]. Patients with CHD usually have a very large annulus associated with degenerative involvement of the TV leaflets and subvalvular chordae. Therefore, the restriction of the annulus does not completely correct the TV vice [75,76]. The purpose of heterotopic implantation of valves in the cava is to prevent systemic venous congestion and blood reflux in the advanced stage of the disease [77]. Before the procedure, it is important to measure the diameters of both superior and inferior cava veins at the level of cavo-atrial junctions, by CT scan. Additionally, significant backflow in the inferior vena cava and/or superior vena cava, with a v-wave ≥25 mmHg has to be demonstrated by right heart catheterization. If the right atrium is too dilatated, the risk of the migration of the valve is increased because the cavo-atrial junction has a particular shape. Moreover, it is critical to confirm that the RV function is preserved or at normal lower limits because after the positioning of caval implants, the right atrial pressure and volume increase, so adverse RV remodeling may occur without symptomatic relief [77]. Although there are few contraindications anatomically, this intervention does not directly address the TV but merely palliates the effects of severe TR. This poses a risk of further impairing stroke volume, right ventricular function, and overall hemodynamics in these already critical patients [78]. Finally, transcatheter TV replacement may be considered a valuable option. Still, the risk of bleeding due to the need for adequate anticoagulation after prosthesis implantation may represent the most important issue in this setting of patients, along with the risk of post-procedural afterload mismatch [78]. Emerging transcatheter therapeutic approaches for tricuspid regurgitation could overcome some of the anatomical challenges posed by carcinoid involvement of the tricuspid valve [79,80].

## 9. Conclusions

Despite well-established algorithms for the screening, diagnosis, and follow-up of CHD, selecting the appropriate surgical or transcatheter management for right-sided valvular disease remains challenging. The complexity of these patients, due to the underlying tumor activity and heart involvement, requires careful patient selection and tailored treatment strategies. As TTVI approaches evolve, they offer new possibilities for patients who are not suitable candidates for traditional surgery. However, further studies and more detailed data are required to optimize treatment outcomes in this specific subgroup of patients with CHD, ensuring the best therapeutic approach for both short- and long-term success.

## Abbreviations

N-terminal pro–B-type natriuretic peptide (NT-proBNP), 5-hydroxyindoleacetic acid (5-HIAA), 5-hydroxytryptamine (5-HT), carcinoid heart disease (CHD), cardiac magnetic resonance (CMR), chromogranin A (CgA), computed tomography (CT), transthoracic echocardiogram (TTE), neuroendocrine tumor (NET), peptide receptor radionuclide treatment (PRRT), pulmonary valve (PV), soluble suppression of tumorigenesis-2 (sST2), transthoracic echocardiogram (TTE), right heart failure (RHF), right ventricle (RV), tricuspid valve (TV).
